# AdapterRemoval v2: rapid adapter trimming, identification, and read merging

**DOI:** 10.1186/s13104-016-1900-2

**Published:** 2016-02-12

**Authors:** Mikkel Schubert, Stinus Lindgreen, Ludovic Orlando

**Affiliations:** Centre for GeoGenetics, Natural History Museum of Denmark, University of Copenhagen, 1350 Copenhagen, Denmark; Department of Biology, Section for Computational and RNA Biology, University of Copenhagen, Ole Maaloes Vej 5, 2200 Copenhagen, Denmark; Carlsberg Research Laboratory, Gamle Carlsberg Vej 4-10, 1799 Copenhagen, Denmark; Laboratoire AMIS, Université de Toulouse, University Paul Sabatier (UPS), CNRS UMR 5288, 37 Allées Jules Guesde, 31000 Toulouse, France

**Keywords:** Adapter identification, Adapter trimming, Data pre-processing, High-throughput sequencing, Sequence alignment

## Abstract

**Background:**

As high-throughput sequencing platforms produce longer and longer reads, sequences generated from short inserts, such as those obtained from fossil and degraded material, are increasingly expected to contain adapter sequences. Efficient adapter trimming algorithms are also needed to process the growing amount of data generated per sequencing run.

**Findings:**

We introduce AdapterRemoval v2, a major revision of AdapterRemoval v1, which introduces *(i)* striking improvements in throughput, through the use of single instruction, multiple data (SIMD; SSE1 and SSE2) instructions and multi-threading support, *(ii)* the ability to handle datasets containing reads or read-pairs with different adapters or adapter pairs, *(iii)* simultaneous demultiplexing and adapter trimming, *(iv)* the ability to reconstruct adapter sequences from paired-end reads for poorly documented data sets, and *(v)* native gzip and bzip2 support.

**Conclusions:**

We show that AdapterRemoval v2 compares favorably with existing tools, while offering superior throughput to most alternatives examined here, both for single and multi-threaded operations.

**Electronic supplementary material:**

The online version of this article (doi:10.1186/s13104-016-1900-2) contains supplementary material, which is available to authorized users.

## Findings

### Background

High-throughput sequencing of short DNA fragments, such as those produced from fossil material [1], may result in the sequencing of the adapter sequences that have been ligated to inserts during library preparation. Such contamination is a well-known problem and may negatively impact downstream analyses [2–5]. The first part of the workflow therefore typically includes a step to filter or remove (trim) adapter contamination [3]. Improved fidelity may furthermore be obtained from paired-end sequencing of short inserts by detecting overlapping reads and collapsing (merging) these in a quality-aware fashion to reconstruct the entire template molecule [6]. This is of particular interest to ancient DNA sequencing, where short inserts are expected, and where decreasing the already high rates of sequencing errors, caused by *post*-*mortem* DNA modifications towards read termini, is of interest [7].

The original release of AdapterRemoval v1 [2] offered a user-friendly tool for trimming of adapter sequences and low-quality bases, using a modified version of the Needleman–Wunsch algorithm, in order to perform pair-wise alignment between reads (or read pairs) and known adapter sequences. In the case of single-end reads, this alignment is carried out in a straight-forward manner, by finding the best alignment between the 5′ termini of the adapter sequence and the 3′ termini of the raw sequencing reads, and removing the aligned sequence. In the case of paired-end reads, an alignment is carried out between the mate 1 read and the reverse complement of the mate 2 read, after prefixing the reverse complemented mate 2 adapter sequence prefixed to the mate 1 sequence, and after appending the mate 1 adapter sequence to the reverse complemented mate 2 sequence.

Pairwise alignment of these aggregate sequences allow for the identification of the 3′ termini of the insert sequence in each read, based on the location of the 5′ termini in the other mate sequence, thereby allowing the extraneous (adapter) sequence to be trimmed [2]. However, as the modified Needleman–Wunsch algorithm used does not account for indels introduced by the sequencing technology employed, AdapterRemoval is primarily suited for the processing of reads generated using Illumina HTS platforms, which are characterized by low rates of spurious indels. AdapterRemoval furthermore uses the overlapping fragments detected as part of the adapter alignment procedure, in order to (optionally) merge overlapping reads. This is accomplished by selecting the highest quality bases, and re-calculating base qualities by treating the quality scores at overlapping positions as position specific scoring matrices, from which updated qualities can be obtained [2].

However, AdapterRemoval v1 is characterized by relatively slow running times compared to other modern tools [8], which poorly accommodates the increasing throughput of sequencing platforms. We therefore carried out extensive revisions of AdapterRemoval v1, with the goal of improving throughput, without modifying the trimming methodology employed. The updated version therefore shows an accuracy similar to AdapterRemoval v1, and provides a suitable drop-in replacement for use in existing analytical pipelines [7].

The resulting AdapterRemoval v2 introduces significant improvements in throughput, in part through the use of single instruction, multiple data (SIMD) instructions (namely SSE and SSE2 instructions, commonly supported by consumer-grade CPUs) to accelerate the alignment algorithm used in AdapterRemoval v1, with the added ability to further increase throughput of all operations through the use of multiple threads. In addition, AdapterRemoval v2 allows for the simultaneous trimming of multiple different (pairs of) adapter sequences, selecting the best match per read (pair), and can furthermore transparently read and write gzip and bzip2 compressed FASTQ files. AdapterRemoval v2 further allows for simultaneous demultiplexing and adapter trimming, using a simple maximum-number-of-mismatches comparison for the provided barcodes, and correctly trims paired-end reads extending past the end of the insert. Such reads are terminated by the reverse-complemented barcode sequence of the other mate followed by an adapter sequence, both of which must be removed. Finally, AdapterRemoval v2 can reconstruct putative adapter sequences from overlapping read pairs, which can be used to detect experimental errors and help analyze poorly documented data sets.

To evaluate the performance of AdapterRemoval v2, we compared it with AdapterRemoval v1, and with a selection of contemporary adapter trimming software, in terms of the ability to correctly trim adapter sequences, and in terms of throughput when using one or more threads (where applicable). We further compared the ability of AdapterRemoval to correctly merge overlapping sequences, with several other programs. For the latter, we restricted our comparison to programs which are designed to carry out read merging in the presence of adapter contamination, but note the existence of several alternatives which are well suited for datasets containing little or no adapter contamination, *e.g.* COPE [9], fastq-join [10], FLASH [11], and XORRO (arXiv:1304.4620).

### Methods

We compared AdapterRemoval v2.1.3 with AdapterRemoval v1.5.4 [2], using parameters equivalent to the defaults for AdapterRemoval v2.x (--mm 3 for both single-end and paired-end reads); with AlienTrimmer v0.4.0 [4], using the natively compiled version, see below; with CutAdapt v1.8.3 [12]; with leeHom rev. dfca9e6 [13], with and without the '--ancientdna' option; with PEAR v0.9.6 [14]; with PEAT rev. 4e9ebf3 [14]; with fastq-mcf v1.1.2 (https://code.google.com/p/ea-utils/); with FLEXBAR v2.5 [15], pre-compiled version; with Scythe v0.991 (https://github.com/ucdavis-bioinformatics/scythe); with Skewer v0.1.127 [16]; and with Trimmomatic v0.33 [5]. We furthermore compared AdapterRemoval v2.1.3 with Minion from the Kraken suite of tools [17]. Software was compiled with GCC v4.8.4 on the target machine, with the exception of Minion, Trimmomatic, and flexbar, for which pre-compiled versions were used, and AlienTrimmer was compiled using GCJ v4.8.4. Trimmomatic was executed using the Oracle JRE v1.8.0, update 66.

For the purpose of comparing running times, we disabled compression of output files. For applicable programs, we disabled trimming of low-quality bases, and any minimum length requirements for the trimmed sequences, in order to avoid measuring the removal of bases not related to the adapter trimming algorithms. We caution that the latter may negatively impact the sensitivity of AlienTrimmer, for which recommended usage includes trimming of low-quality bases (Phred <20), prior to the detection of adapter sequences.

For benchmarking adapter trimming, we defined true positives (TP) as reads trimmed to the expected length; true negatives (TN) as reads not containing adapters left untrimmed; false positives (FP) as reads trimmed for more bases than expected; and false negatives (FN) as reads which still contained adapter bases post trimming. For benchmarking read merging we defined as True Positives those overlapping reads that were merged to the expected length; true negatives as non-overlapping reads which were not merged; false positives as non-overlapping reads which were merged and overlapping reads merged incorrectly; and false negatives as overlapping reads that were not merged. Throughput is reported as the average number of sequences processed per second, in thousands, counting both mates in paired-end read-pairs as individual reads.

We summarize these results following Lindgreen 2012 [2]; namely by sensitivity [*SEN* = *TP/(TP* + *FN)]*, specificity [SPC = TN/(FP + TN)], positive predictive value [PPV = TP/(TP + FP)], negative predictive value [NPV = TN/(TN + FN)], and, as an overall measure of the performance, the Matthews correlation coefficient {MCC = (TP × TN–FP × FN)/√[(TP + FP) × (TP + FN) × (TN + FP) × (TN + FN)]}.

We simulated 10 replications of 1 million 100 bp paired-end reads, with a mean insert size of 150 bp, and a standard deviation of 75 bp, using a modified version of pIRS v1.1.1 [18]. This version had been modified such that insert sizes less than the read length were allowed, and that adapter sequences were appended to such inserts prior to the simulation of read errors. We used single indexed Illumina HISeq adapter sequences, “AGATC GGAAG AGCAC ACGTC TGAAC TCCAG TCACN NNNNN ATCTC GTATG CCGTC TTCTG CTTG” and “AGATC GGAAG AGCGT CGTGT AGGGA AAGAG TGTAG ATCTC GGTGG TCGCC GTATC ATT”, during read simulations. Reads were simulated against the human chromosome 1 sequence, using the hg38 reference genome.

All results are reported as the average obtained from trimming each of the 10 replicate datasets; the order in which programs were run in each replicate was randomized. To benchmark throughput, we furthermore simulated 200 bp long reads. In the absence of a biological read profile, we duplicated each position in the 100 bp default error-profiles for pIRS v1.1.1, and simulated 200 bp long reads using this profile, with a mean insert-size of 300 bp, and a standard deviation of 100 bp. As these error profiles are not representative, we only used these to examine data throughput.

For benchmarking trimming of multiple adapters, we generated four additional pairs of adapters by shuffling the nucleotides in the adapters listed above, for a total of five pairs of adapter sequences. We next generated 1 M reads as described previously, and randomly selected an adapter pair for each insert. Trimming performance was measured as described above.

For benchmarking of adapter sequence identification, we shuffled the sequence of the default adapter-pair for AdapterRemoval, and generated 1 million reads as described above using the shuffled adapter pair, a variable mean insert size with a standard deviation of 75 bp. We counted the number of correctly called bases from the 5′ end of the resulting adapter sequences (Fig. 3). For Minion, we considered the five best sequences for each run, and selected the best match.

Benchmarking was carried out on an otherwise idle Intel^®^ Core™ i7-4790 K 4 × 4.00 GHz, with 8 GB of DDR3-2133 RAM, on an ext4 partition on a Samsung SSD 840 EVO 750 GB drive. The scripts used for the performance tests described in this paper, as well as any patches applied to programs used in the tests, are stored in the AdapterRemoval v2 GitHub repository in the ‘benchmark’ folder.

### Results and discussion

**Fig. 1:**
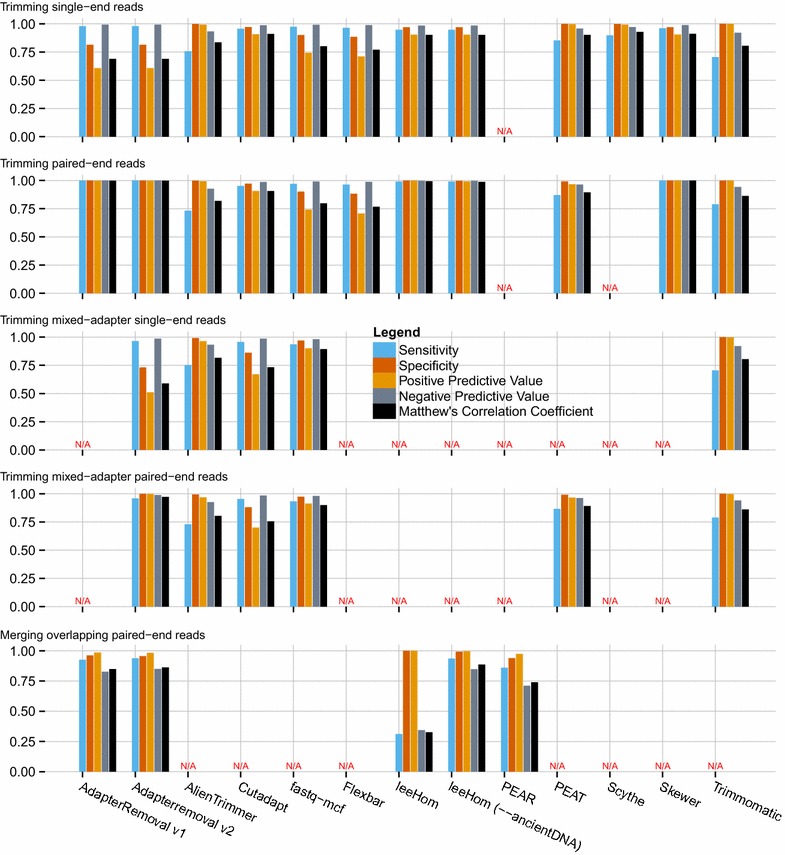
Adapter-trimming and read-merging performance. Performance metrics for trimming of single adapter-pairs, multiple adapter-pairs, and merging of overlapping read pairs

The performance of AdapterRemoval v2 was compared to a selection of other adapter trimming software, as described above (Fig. 1 and Additional File 1: Table S1), for both single-end and paired-end operations, as well as for merging of overlapping paired-end reads.

AdapterRemoval v2 was found to offer high sensitivity (0.979), at the cost of lower specificity (0.814) when trimming single-end data sets containing either a single adapter sequence, or containing multiple adapter sequences. Similar high sensitivity (0.965) low specificity (0.731) was observed for data sets containing multiple, different adapters. This is a consequence of AdapterRemoval not requiring a minimum length of the overlap with the adapter sequence, resulting in an excess of short (1–3 bp) fragments trimmed from the 3′ termini of single-end reads. This behavior can, however, be changed by use of the '--minadapteroverlap' command-line option (e.g. '--minadapteroverlap 3'), and secondarily by decreasing the allowed error-rate (e.g. by using '--mm 5' rather than '--mm 3', corresponding to an error rate of 1/5 rather than 1/3). When run using these parameters, AdapterRemoval shows levels of specificity comparable to other tools (0.962 and 0.972), at the cost of a marginal decrease in sensitivity (0.959 and 0.956), when trimming data sets containing a single adapter sequence. For data sets containing multiple adapter sets, these parameters similarly led to a marginal decrease in sensitivity (0.960 and 0.965) and an increase in specificity (0.817 and 0.858).

However, AdapterRemoval v2 displays both high sensitivity (0.999) and specificity (0.999) when considering paired-end data, when using default parameters. High sensitivity (0.959) and specificity (0.999) was also observed for data sets containing multiple adapter pairs. For merging of overlapping pair-end reads, AdapterRemoval offers comparable similar sensitivity (0.938) and specificity (0.955) to the alternatives examined here.

**Fig. 2:**
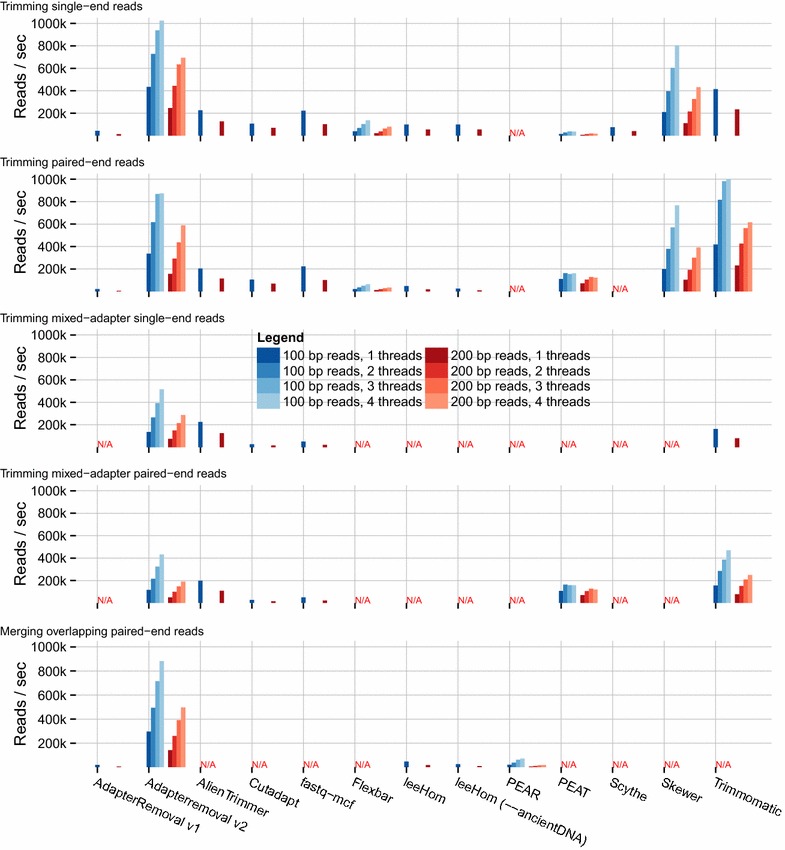
Adapter-trimming and read-merging throughput. Throughput is reported on the y-axis as thousands of FASTQ reads processed per second. Results are grouped on the x-axis firstly per program, secondly by read length (100 or 200 bp), and thirdly by the number of threads used (1–4). For programs that do not support multi-threaded operation, only columns corresponding to one thread are shown. Note that multi-threaded trimming of single-end reads using Trimmomatic was excluded, due to erratic behavior on the test machine. Benchmarking was carried out on an otherwise idle Intel^®^ Core™ i7-4790 K 4 × 4.00 GHz, with 8 GB of DDR3-2133 RAM, on an ext4 partition on a Samsung SSD 840 EVO 750 GB drive

When comparing the throughput of each program (Fig. 2 and Additional File 2: Table S2), we observed that AdapterRemoval v2 offers the highest throughput next to Trimmomatic, for the trimming of 100 bp single-end (434 vs 414 k reads/sec) and paired-end (336 k vs 418 k reads/sec) reads, and scales well with multi-threading. For the merging of overlapping reads in the presence of adapter sequences, AdapterRemoval greatly outperforms all alternatives by an order of magnitude (295 vs 47 k reads/sec for leeHom). For trimming of data sets containing multiple adapter sequences, AdapterRemoval was out-performed by AlienTrimmer and Trimmomatic, for both single-end reads (136 vs 225 and 162 k reads/sec) and paired-end reads (117 vs 198 and 156 k reads/sec), respectively. AdapterRemoval v2 is therefore particularly well suited for the processing of large data sets, enabling the processing of large amounts of data on a desktop machine. Furthermore, performance scales well for increasing read lengths, ensuring that AdapterRemoval v2 is suitable for use with the progressively longer read-lengths generated by high-throughput sequencing platforms.

**Fig. 3:**
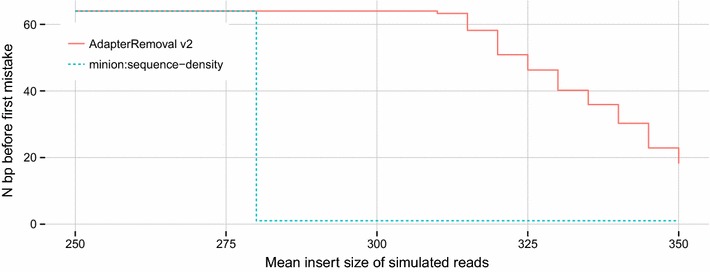
Fidelity of adapter sequence reconstruction from paired-end reads. The *x-axis* represents the mean insert size of simulated, paired-end reads in bp, with a standard deviation of 75 bp. The *y-axis* represents the number of bases before first mistake in the (best) recovered adapter sequence for the mate 1 adapter sequence

The ability of AdapterRemoval v2 to reconstruct unknown adapter sequences compared to Minion is shown in Fig. 3. Minion is able to perfectly recover the randomized adapter sequence for mean insert sizes below ~280 bp, but fails entirely for greater mean insert sizes. AdapterRemoval v2 recovers the complete adapter sequence for insert size means up to ~300 bp, and partially recovers the adapter sequence for greater mean insert sizes, potentially allowing for the identification of the original adapter from published vendor sequences.

However, we note that while AdapterRemoval v2 outperforms Minion when analyzing paired-end reads, Minion can be used to identify adapter sequences in single-end reads, which is not possible using AdapterRemoval v2. Minion is furthermore able to identify multiple, overrepresented sequences, while AdapterRemoval v2 makes use of consensus building from putative adapter sequences, resulting in poor performance should multiple, different adapter sequences be present.

## Availability and requirements

- *Project name*: AdapterRemoval
- *Project home page*: https://github.com/MikkelSchubert/adapterremoval/
- *Operating system(s)*: POSIX (tested on Linux and OSX)
- *Programming language*: C++
- *Other requirements*: Optional support for gzip compression, bzip2 compression, and multi-threading requires zlib, libbzip2, and libpthreads, respectively
- *License*: GPL v3.

## Additional files

10.1186/s13104-016-1900-2 Adapter-trimming and read-merging performance. Tabular representation of performance metrics for trimming of single adapter-pairs, multiple adapter-pairs, and merging of overlapping read pairs (Fig. 1).
10.1186/s13104-016-1900-2 Adapter-trimming and read-merging throughput. Tabular representation of throughput of adapter trimming and read merging reported as thousands of FASTQ reads processed per second (Fig. 2).
