# Supplementary material for: AdapterRemoval v2: rapid adapter trimming, identification, and read merging
Source: BMC Res Notes. 2016 Feb 12;9:88. doi: 10.1186/s13104-016-1900-2 (PMC4751634; doi:10.1186/s13104-016-1900-2)
Supplement: Supplementary file 1 — 10.1186/s13104-016-1900-2 Adapter-trimming and read-merging performance. Tabular representation of performance metrics for trimming of single adapter-pairs, multiple adapter-pairs, and merging of overlapping read pairs (Fig. 1). [file 13104_2016_1900_MOESM1_ESM.docx]

**Supplementary Table S1. Adapter-trimming and read-merging performance**

| **Trimming of SE reads** | **SEN** | **SPC** | **PPV** | **NPV** |  | **MCC** |
| --- | --- | --- | --- | --- | --- | --- |
| AdapterRemoval v1 | 0.979 | 0.814 | 0.607 | 0.992 |  | 0.690 |
| AdapterRemoval v2 | 0.979 | 0.814 | 0.607 | 0.992 |  | 0.690 |
| --minadapteroverlap 3 | 0.959 | 0.962 | 0.881 | 0.998 |  | 0.895 |
| --minadapteroverlap 3 --mm 5 | 0.956 | 0.972 | 0.910 | 0.987 |  | 0.913 |
| AlienTrimmer | 0.757 | 0.998 | 0.993 | 0.933 |  | 0.836 |
| Cutadapt | 0.956 | 0.971 | 0.907 | 0.987 |  | 0.910 |
| Fastq-mcf | 0.974 | 0.901 | 0.743 | 0.991 |  | 0.801 |
| Flexbar | 0.964 | 0.885 | 0.711 | 0.988 |  | 0.771 |
| leeHom | 0.947 | 0.970 | 0.903 | 0.984 |  | 0.902 |
| leeHom (--ancientDNA) | 0.947 | 0.970 | 0.903 | 0.984 |  | 0.902 |
| PEAT | 0.853 | 0.999 | 0.997 | 0.958 |  | 0.902 |
| Scythe | 0.898 | 0.998 | 0.992 | 0.971 |  | 0.929 |
| Skewer | 0.960 | 0.970 | 0.905 | 0.988 |  | 0.911 |
| Trimmomatic | 0.705 | 1.000 | 0.999 | 0.920 |  | 0.805 |
|  |  |  |  |  |  |  |
| **Trimming of PE reads** | **SEN** | **SPC** | **PPV** | **NPV** |  | **MCC** |
| AdapterRemoval v1 | 0.999 | 0.999 | 0.998 | 1.000 |  | 0.998 |
| AdapterRemoval v2 | 0.999 | 0.999 | 0.998 | 1.000 |  | 0.998 |
| AlienTrimmer | 0.732 | 0.998 | 0.993 | 0.927 |  | 0.819 |
| Cutadapt | 0.952 | 0.971 | 0.906 | 0.986 |  | 0.907 |
| Fastq-mcf | 0.970 | 0.901 | 0.742 | 0.990 |  | 0.798 |
| Flexbar | 0.964 | 0.883 | 0.707 | 0.988 |  | 0.767 |
| leeHom | 0.990 | 1.000 | 1.000 | 0.997 |  | 0.993 |
| leeHom (--ancientDNA) | 0.990 | 0.997 | 0.991 | 0.997 |  | 0.988 |
| PEAT | 0.871 | 0.991 | 0.966 | 0.963 |  | 0.895 |
| Skewer | 0.998 | 1.000 | 1.000 | 0.999 |  | 0.999 |
| Trimmomatic | 0.790 | 1.000 | 0.999 | 0.942 |  | 0.862 |
|  |  |  |  |  |  |  |
| **Trimming of mixed SE reads** | **SEN** | **SPC** | **PPV** | **NPV** |  | **MCC** |
| AdapterRemoval v2 | 0.965 | 0.731 | 0.511 | 0.986 |  | 0.589 |
| --minadapteroverlap 3 | 0.960 | 0.817 | 0.604 | 0.986 |  | 0.677 |
| --minadapteroverlap 3 --mm 5 | 0.965 | 0.858 | 0.664 | 0.985 |  | 0.727 |
| AlienTrimmer | 0.753 | 0.992 | 0.964 | 0.932 |  | 0.817 |
| Cutadapt | 0.958 | 0.861 | 0.670 | 0.986 |  | 0.733 |
| Fastq-mcf | 0.936 | 0.969 | 0.900 | 0.981 |  | 0.893 |
| Trimmomatic | 0.705 | 1.000 | 0.999 | 0.920 |  | 0.805 |
|  |  |  |  |  |  |  |
| **Trimming of mixed PE reads** | **SEN** | **SPC** | **PPV** | **NPV** |  | **MCC** |
| AdapterRemoval v2 | 0.959 | 0.999 | 0.998 | 0.988 |  | 0.972 |
| AlienTrimmer | 0.729 | 0.993 | 0.968 | 0.926 |  | 0.803 |
| Cutadapt | 0.954 | 0.880 | 0.700 | 0.985 |  | 0.756 |
| Fastq-mcf | 0.933 | 0.973 | 0.912 | 0.980 |  | 0.899 |
| PEAT | 0.866 | 0.991 | 0.966 | 0.962 |  | 0.891 |
| Trimmomatic | 0.789 | 1.000 | 0.998 | 0.941 |  | 0.861 |
|  |  |  |  |  |  |  |
| **Merging of overlapping read pairs** | **SEN** | **SPC** | **PPV** | **NPV** |  | **MCC** |
| AdapterRemoval v1 | 0.926 | 0.962 | 0.985 | 0.826 |  | 0.849 |
| AdapterRemoval v2 | 0.938 | 0.955 | 0.983 | 0.850 |  | 0.863 |
| leeHom | 0.312 | 1.000 | 1.000 | 0.344 |  | 0.327 |
| leeHom (--ancientDNA) | 0.936 | 0.993 | 0.997 | 0.848 |  | 0.886 |
| PEAR | 0.860 | 0.939 | 0.974 | 0.711 |  | 0.740 |

Performance metrics for trimming of single adapter-pairs, multiple adapter-pairs, and merging of overlapping read pairs. SEN=sensitivity, SPC=specificity, PPV=positive predictive value, NPV=negative predictive value, MCC=Matthew’s correlation coefficient.
