# Supplementary material for: AdapterRemoval v2: rapid adapter trimming, identification, and read merging
Source: BMC Res Notes. 2016 Feb 12;9:88. doi: 10.1186/s13104-016-1900-2 (PMC4751634; doi:10.1186/s13104-016-1900-2)
Supplement: Supplementary file 2 — 10.1186/s13104-016-1900-2 Adapter-trimming and read-merging throughput. Tabular representation of throughput of adapter trimming and read merging reported as thousands of FASTQ reads processed per second (Fig. 2). [file 13104_2016_1900_MOESM2_ESM.docx]

**Supplementary Table S2. Adapter-trimming and read-merging throughput**

| **Trimming of SE reads** | **100/1** | **100/2** | **100/3** | **100/4** | **200/1** | **200/2** | **200/3** | **200/4** |
| --- | --- | --- | --- | --- | --- | --- | --- | --- |
| AdapterRemoval v1 | 43.2 |  |  |  | 12.8 |  |  |  |
| AdapterRemoval v2 | 434.4 | 728.3 | 938.2 | 1024.0 | 246.3 | 443.1 | 634.1 | 693.5 |
| --minadapteroverlap 3 | 435.6 |  |  |  | 239.2 |  |  |  |
| --minadapteroverlap 3 --mm 5 | 435.6 |  |  |  | 244.4 |  |  |  |
| AlienTrimmer | 225.9 |  |  |  | 127.7 |  |  |  |
| Cutadapt | 106.4 |  |  |  | 69.6 |  |  |  |
| Fastq-mcf | 221.9 |  |  |  | 101.4 |  |  |  |
| Flexbar | 38.6 | 68.1 | 103.2 | 136.1 | 22.1 | 37.5 | 62.8 | 78.6 |
| leeHom | 99.3 |  |  |  | 55.1 |  |  |  |
| leeHom (--ancient) | 99.3 |  |  |  | 55.2 |  |  |  |
| PEAT | 14.6 | 27.7 | 36.9 | 36.2 | 6.9 | 13.2 | 19.3 | 17.5 |
| Scythe | 75.1 |  |  |  | 40.0 |  |  |  |
| Skewer | 210.3 | 395.9 | 604.3 | 805.1 | 111.0 | 214.9 | 325.6 | 431.0 |
| Trimmomatic | 413.9 | * | * | * | 233.5 | * | * | * |
|  |  |  |  |  |  |  |  |  |
| **Trimming of PE reads** | **100/1** | **100/2** | **100/3** | **100/4** | **200/1** | **200/2** | **200/3** | **200/4** |
| AdapterRemoval v1 | 21.8 |  |  |  | 5.3 |  |  |  |
| AdapterRemoval v2 | 336.2 | 617.7 | 868.6 | 873.8 | 156.1 | 292.1 | 436.7 | 588.8 |
| AlienTrimmer | 204.1 |  |  |  | 114.3 |  |  |  |
| Cutadapt | 105.0 |  |  |  | 69.2 |  |  |  |
| Fastq-mcf | 223.6 |  |  |  | 101.3 |  |  |  |
| Flexbar | 21.3 | 36.0 | 50.1 | 64.3 | 11.8 | 20.5 | 27.6 | 34.6 |
| leeHom | 47.4 |  |  |  | 18.5 |  |  |  |
| leeHom (--ancient) | 24.8 |  |  |  | 8.8 |  |  |  |
| peat | 111.0 | 162.9 | 155.3 | 161.3 | 71.6 | 104.9 | 127.6 | 122.5 |
| Skewer | 199.2 | 379.9 | 570.3 | 767.4 | 103.9 | 191.9 | 299.8 | 390.8 |
| Trimmomatic | 417.6 | 816.2 | 981.9 | 1001.3 | 240.4 | 425.5 | 563.9 | 615.2 |
|  |  |  |  |  |  |  |  |  |
| **Trimming of mixed SE reads** | **100/1** | **100/2** | **100/3** | **100/4** | **200/1** | **200/2** | **200/3** | **200/4** |
| AdapterRemoval v2 | 135.7 | 266.1 | 392.6 | 515.7 | 74.8 | 149.1 | 214.7 | 286.6 |
| --minadapteroverlap 3 | 136.9 |  |  |  | 75.5 |  |  |  |
| --minadapteroverlap 3 --mm 5 | 137.0 |  |  |  | 75.4 |  |  |  |
| AlienTrimmer | 225.4 |  |  |  | 125.2 |  |  |  |
| Cutadapt | 26.0 |  |  |  | 15.2 |  |  |  |
| Fastq-mcf | 50.0 |  |  |  | 21.4 |  |  |  |
| Trimmomatic | 162.2 | * | * | * | 79.3 | * | * | * |
|  |  |  |  |  |  |  |  |  |
| **Trimming of mixed PE reads** | **100/1** | **100/2** | **100/3** | **100/4** | **200/1** | **200/2** | **200/3** | **200/4** |
| AdapterRemoval v2 | 117.2 | 216.1 | 323.4 | 432.5 | 50.8 | 99.8 | 147.7 | 190.2 |
| AlienTrimmer | 198.2 |  |  |  | 109.4 |  |  |  |
| Cutadapt | 26.8 |  |  |  | 15.6 |  |  |  |
| Fastq-mcf | 50.4 |  |  |  | 21.9 |  |  |  |
| PEAT | 107.8 | 163.0 | 158.6 | 158.7 | 69.9 | 106.7 | 127.6 | 119.5 |
| Trimmomatic | 156.1 | 285.4 | 386.2 | 469.4 | 77.9 | 151.8 | 208.6 | 249.8 |
|  |  |  |  |  |  |  |  |  |
| **Merging of overlapping read pairs** | **100/1** | **100/2** | **100/3** | **100/4** | **200/1** | **200/2** | **200/3** | **200/4** |
| AdapterRemoval v1 | 19.6 |  |  |  | 5.0 |  |  |  |
| AdapterRemoval v2 | 295.4 | 493.8 | 715.3 | 881.7 | 141.4 | 260.5 | 390.3 | 496.6 |
| leeHom | 47.1 |  |  |  | 18.5 |  |  |  |
| leeHom (--ancient) | 24.8 |  |  |  | 8.8 |  |  |  |
| PEAR | 20.6 | 38.3 | 61.6 | 71.3 | 5.3 | 10.5 | 14.8 | 17.9 |

Throughput is reported as thousands of FASTQ reads processed per second. Empty cells indicate that a run was not carried out due to lack of support for multi-threaded analyses for a given program. Read sizes (100 or 200 bp) and the number of threads used (1 to 4) are indicated before and after the slashes, respectively. * Multi-threaded trimming of SE reads using Trimmomatic was excluded, due to erratic behavior on the test machine.
